# Adaptation and validation of two annotation scales for assessing social skills in a corpus of multimodal collaborative interactions

**DOI:** 10.3389/fpsyg.2022.1039169

**Published:** 2022-10-27

**Authors:** Jennifer Hamet Bagnou, Elise Prigent, Jean-Claude Martin, Céline Clavel

**Affiliations:** Université Paris-Saclay, CNRS, Laboratoire Interdisciplinaire des Sciences du Numérique (LISN), Orsay, France

**Keywords:** social skills, collaboration, problem solving, annotation, scale validation, social interaction, dyadic interaction, video corpus

## Abstract

**Context:**

Behavioral observation scales are important for understanding and assessing social skills. In the context of collaborative problem-solving (CPS) skills, considered essential in the 21st century, there are no validated scales in French that can be adapted to different CPS tasks. The aim of this study is to adapt and validate, by annotating a new video corpus of dyadic interactions that we have collected, two observational scales allowing us to qualitatively assess CPS skills: the Social Performance Rating Scale (SPRS) and the Social Skills of Collaboration Scale (SSC).

**Method:**

The construct validity of these two scales was assessed by exploratory factor analysis and inter-item correlations. We also checked inter-judge agreement using inter-class correlation coefficients. Internal consistency was determined using Cronbach’s alpha and convergent and divergent validity by assessing correlations between the two scales and measures of depression and alexithymia. Finally, the discriminative properties of the two scales were analyzed by comparing the scores obtained by a group of anxious individuals and a non-anxious control group.

**Results:**

The results show that our two scales have excellent inter-item correlations. Internal consistency is excellent (alpha SPRS =0.90; SSC = 0.93). Inter-rater agreement ranged from moderate to high. Finally, convergent validity was significant with the alexithymia scale, as was divergent validity with the depression scale. Anxious individuals had lower scores on both scales than non-anxious individuals.

**Conclusion:**

Both scales show good psychometric properties for assessing social skills relevant to different collaborative tasks. They also identify individuals with difficulties in social interaction. Thus, they could allow monitoring the effectiveness of training social skills useful in CPS.

## Introduction

Collaborative problem-solving (CPS) is a form of interaction that contributes significantly to the functioning of a group or organization (Aram et al., 1971). It is defined as a set of joint social skills to solve problems and work toward a common goal while socially collaborating with each other in a group of individuals (O'Neil et al., 2010; Hesse et al., 2015; Stadler et al., 2020). Thus, CPS depends on the ability of individuals to establish common ground regarding the nature of the problem, develop a solution plan, monitor progress along the way, and consider multiple viewpoints while respectfully managing disagreements. This requires the ability to understand the goals and constraints of the task and to consider the perspectives and knowledge of others, as well as the ability to communicate this understanding through negotiation, mutual regulation, and shared responsibility (Sun et al., 2020).

Recent research has identified CPS skills as critical to academic and career success (Andrews-Todd and Forsyth, 2020). Indeed, as individuals enter the workforce, they are expected to work with others to solve complex, non-automatable problems, make decisions, and generate new ideas, which requires skills associated with CPS. Even experienced workers collaborate with colleagues to combine their expertise and find a common solution to a problem.

Beyond work or school, we also collaborate with others to solve problems of various kinds in our private lives, whether with family, friends, or strangers. If we look at our environment, almost all of the objects around us are the product of collaboration (Hutchins, 1995). Their production extends to many fields such as entertainment, health, or even engineering, which is evidence of the necessity of collaboration for progress and development (Hutchins, 1995; Oliveri et al., 2017; Sun et al., 2020).

All of these domains require that the individuals involved have sufficient problem-solving and social collaboration skills (Hesse et al., 2015; Graesser et al., 2018; Stadler et al., 2020). Difficulties in these social skills can therefore create social dysfunction resulting in difficult social interactions with others, social withdrawal, and emotional distress (Bellack, 2004). These dysfunctions are often associated with the absence of the required skills, not using them in a timely manner, or when the individual engages in socially inappropriate behaviors (Bellack, 1983, 2004; Kingery et al., 2020), or individual characteristics (Greene, 2003; Oliveri et al., 2017), such as anxiety level (Greene, 2003; Stevens et al., 2010) or alexithymia (Spitzer and Siebel-Jürges, 2005).

Yet, as observed by Hesse et al. (2015), these complex skills useful during CPS tasks are neither taught nor formally assessed. One reason for this is the lack of consensus on a CPS model to operationalize this construct and measure it (Oliveri et al., 2017; Andrews-Todd et al., 2018; Sun et al., 2020).

Thus, studies of CPS use a variety of measures to assess them (e.g., surveys, computerized tests, observations, think-aloud protocols, and human-chatbot interactions), and the quality of CPS assessments varies considerably.

These existing assessment tools include self-assessments (e.g., VIEW scale, Selby et al., 2004; Creative Problem-Solving Group, 2013) that use Likert scales or forced-choice options, situational judgment tests (e.g., The Teamwork-KSA Test, Stevens and Campion, 1999), and third-party assessments or observation tools.

These self-report tests and situational judgments have many limitations. Indeed, there is a high risk of response bias (social desirability, extreme responses, acquiescence, halo effect; Cheung and Chan, 2002; Zhuang and MacCann, 2008; Oliveri et al., 2017). Participants must have high metacognition skills to judge their own level of performance. These tools can also induce mismatches between the experimenter’s and the participant’s judgment.

As for third-party assessments, existing observation tools are often limited to the health and medical domain (e.g., Communication and Teamwork skills, Frankel and Gardner, 2007) and the military domain (Anti-Air Teamwork Observation Measure; Smith-Jentsch et al., 1998). Several observation tools were developed on the basis of Human-Agent interactions and not between two humans (Dindar et al., 2020; Stadler et al., 2020). Moreover, their psychometric analyses show low inter-rater reliabilities (Oliveri et al., 2017).

All of these validated tools are often used in a higher education admissions context and thus typically cater to a young population, moving from one grade level to the next (Oliveri et al., 2017). Yet, CPS skills are acquired throughout the lifespan (Graesser et al., 2018; Stadler et al., 2020). Added to this is a growing demand from researchers on consensus building for CPS assessment (Andrews-Todd et al., 2018; Bause and Brich, 2018; Dindar et al., 2020; Stadler et al., 2020; Sun et al., 2020).

Thus, the development and validation of qualitative performance measurement scales that are adaptable to different situations and free from response bias would allow for a more standardized assessment of social skills (Dindar et al., 2020; Stadler et al., 2020). Furthermore, it is important to have a reliable, validated, and discriminating tool to verify the effectiveness of skills training and the progression of participants according to their individual characteristics.

In this paper, we present the experimental protocol we have set up to collect social interactions during collaborative games requiring CPS-like skills, as well as the validation of two scales to assess the collaborative and social skills observed there.

To assess social skills during CPS tasks, we built on the framework proposed by ATC21S to create a new scale (Hesse et al., 2015), and we considered an existing annotation scale, Fydrich’s Social Performance Rating Scale (SPRS; Fydrich and Chambless, 1998).

This article is divided into five parts. In the first part, we will describe the corpus collection and the development of the two annotation scales. In the second part, we will present the validation of the two scales with the population, as well as the protocol used for the validation of the two scales. In the third part, we will present the results obtained and in the fourth part, we will discuss them. Finally in the fifth part, we will present the limits of our work as well as the future directions.

## Materials and methods

### Corpus

To conduct research on social skills assessment and training, we adapted two widely studied collaborative games, “Prisoner’s Dilemma” (Bland and Roiser, 2017) and “Survival Task” (Hall and Watson, 1970; Johnson, 1994). We also defined a third collaborative game “Investment in Student Social Service Organization” with less constraining game rules and thus allowing for less framed interactions. In Game #1, “Prisoner’s Dilemma,” we asked a pair of participants to reach a consensus on whether or not to turn in their accomplices in order to reduce their sentence. In Game #2, “Survival Task,” participants had to agree on a list of items needed for survival. We added a rule asking the participants to sort the selected items and choose the most important ones in order to make the collaboration necessary. In Game 3, the pair of participants must make a donation to a social service. They must determine the amount of the joint donation and discuss how to use this amount to improve existing services.

We chose these games not only because they fit the definition of CPS, but also because they do not require additional display such as a blackboard or a graphical user interface and thus facilitate natural multimodal interaction among participants.

**Figure 1:**
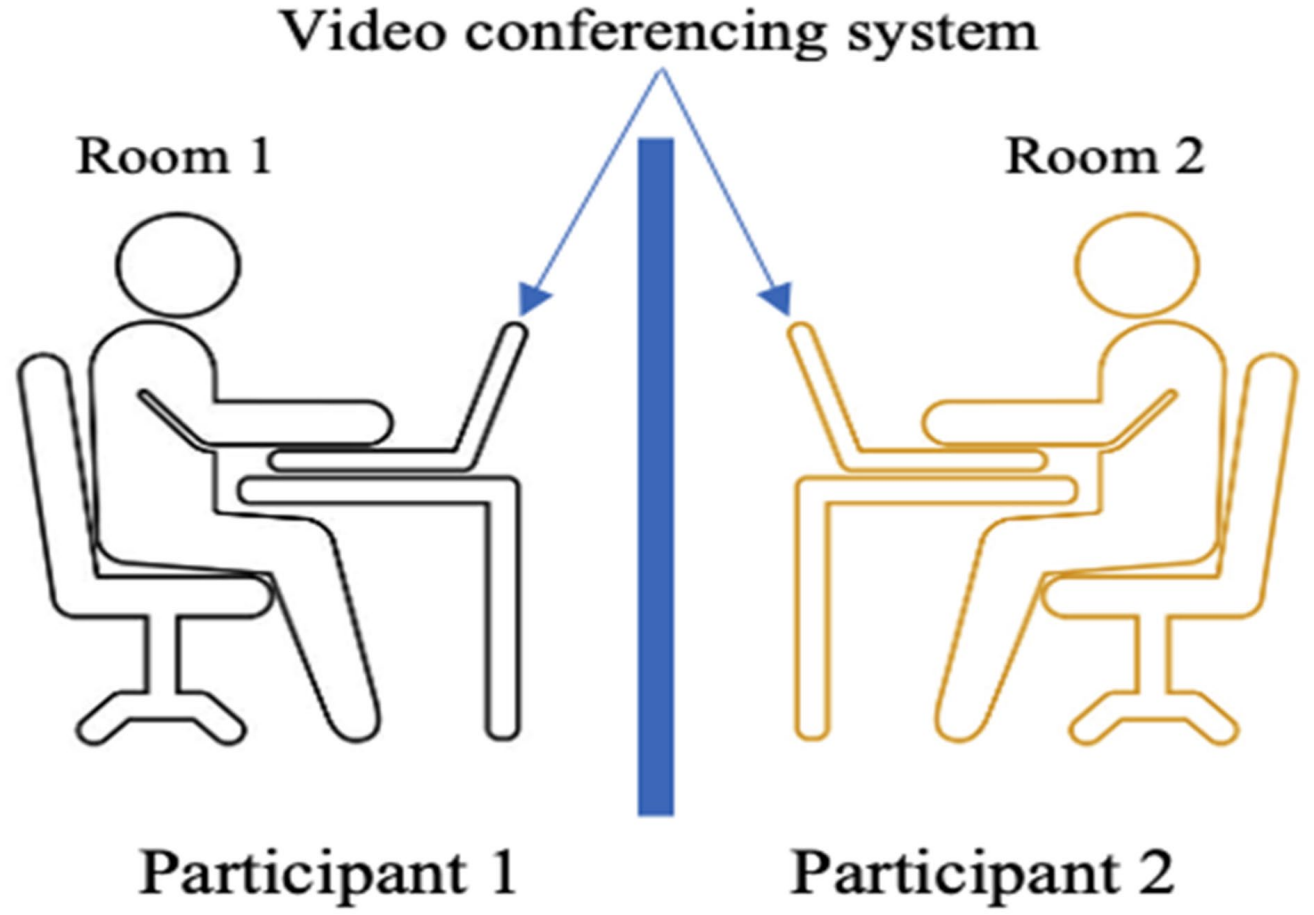
Experimental setup.

Participants were asked to perform the three games *via* a video conferencing system in order to face the COVID situation with remote participation and to control the camera viewpoint at the same time (Figure 1). The order of task completion was randomized.

Each participant was instructed to collaborate with the other participant to find a common solution to a problem within a maximum of 5 min. Participants’ acoustic and visual data were recorded.

Thus, we collected 228 videos and audios with a total of 9 h of recording.

### Scale development phase

#### Design of initial items

The initial items for our third-party CPS Social Skills Annotation Scale were developed based on the theoretical framework defined by the Assessment and Teaching of 21st century skills (ATC21S) project. This framework is based on the distinction between two broad classes of skills: social skills and cognitive skills. Social skills constitute the “collaborative” dimension of collaborative problem solving, while cognitive skills constitute the “problem solving” dimension of collaborative problem solving.

In the problem-solving dimension of CPS, group members work together to develop a shared understanding of the problem situation, exchange information, discuss the most appropriate strategies for solving the problem, and monitor and revise their strategies until the group’s goals are met (Barron, 2003; Zimmerman and Schunk, 2011; Slof et al., 2016). The indicators of these skills can be summarized under two headings: task regulation and knowledge construction. Task regulation refers to learners’ ability to set goals, manage resources, analyze and organize the problem space, explore a problem systematically, gather information, and tolerate ambiguity. Knowledge construction refers to the individual’s ability to understand the problem and test hypotheses.

The “collaborative” dimension includes communication processes among team members that can either facilitate or hinder problem solving (Janssen et al., 2012). Participation, perspective taking, argumentation, negotiation, and emotional and motivational interaction are examples of these communicative processes (Hesse et al., 2015; Slof et al., 2016). Engagement with the task and other collaborators is reflected in how people act or interact to accomplish tasks. Perspective-taking skills focus on the quality of interaction between participants, reflecting their level of awareness of their collaborators’ knowledge and resources as well as their response skills. Social regulation refers to the strategies used in collaboration, such as negotiation, taking initiative, self-evaluation, and taking responsibility.

Thus, we obtain a first annotation grid composed of 18 items divided into 5 domains (3 for the social collaboration dimension and 2 for the problem-solving dimension; see Table 1). We find, for example, items evaluating the level of interaction, the adaptation of the discourse to the other, and the formulation of steps in the problem solving. Each item is evaluated on a scale from 0 (low) to 2 (high).

**Table 1:** Social and cognitive skills in collaborative problem solving, according to ATC21S (Hesse et al., 2015).

Sub-dimension	Element	Description
“Collaborative” dimension		
Participation	Action	Activity within environment
	Interaction	Interacting with, prompting, and responding to the contribution of others
	Task completion/perseverance	Undertaking and completing a task or part of a task individually
Perspective taking	Adaptive responsiveness	Ignoring, accepting, or adapting contributions of others
	Audience awareness	Awareness of how to adapt behavior to increase suitability for others
Social regulation	Negotiation	Achieving a resolution or reaching compromise
	Self-evaluation	Recognizing own strengths and weaknesses
	Transactive memory	Recognizing strengths and weaknesses of others
	Responsibility initiative	Assuming responsibility for ensuring part of task are completed by the group
“Problem solving” dimension		
Task regulation	Organizes	Analyses and describes a problem in familiar language
	Goal sets	Sets a clear goal for a task
	Resource management	Manages resources or people to complete a task
	Flexibility and ambiguity	Accepts ambiguous situations
	Collects elements of information	Explores and understands elements of the task
	Systematicity	lmplements possible solutions to a problem and monitors progress
Learning and knowledge building	Relationship	Identifies connections and patterns between and among elements of knowledge
	Rules “if… then”	Uses understanding of cause and effect to develop a plan
	Hypothesis “what if…”	Adapts reasoning or course of action as information or circumstances change

Unlike other existing models, the one proposed by the ATC21S project lends itself more to the assessment of a human-human interaction and details the sub-skills of collaboration and problem solving. It is also adaptable to different contexts (Hesse et al., 2015; Stadler et al., 2020).

The complexity of CPS requires, in addition to the skills listed above, attention to the verbal and non-verbal communication skills of the social partners (e.g., eye contact, voice quality, etc.). The individual must decide what information is essential to respond, develop a plan, draw on his or her repertoire of verbal and non-verbal skills, and implement it.

Thus, we selected Fydrich’s SPRS (Fydrich and Chambless, 1998; Stevens et al., 2010; Ramdhonee-Dowlot et al., 2021). This scale provides a 5-item qualitative assessment of communication skills in multimodal social interaction. To account for the level of social skills mobilized during social interaction, the authors consider five categories of indicators.

The first indicator involves the flow of conversation which requires behaviors of approaching a social partner, formulating a question or statement, listening to the partner’s response, maintaining the conversation, and ending the interaction (Dotson et al., 2010; Doggett et al., 2013; Chezan et al., 2020). Trower (1980) showed that items in the conversation distinguished socially inadequate psychiatric patients from socially adequate patients (Fydrich and Chambless, 1998). In this item, a very low score will be given to the participant who makes few attempts to initiate conversation, uses almost no open-ended questions, or is intrusive with questions and shows no empathy.

The second indicator concerns voice quality (tone, pitch, clarity, and volume). Indeed, a flat and monotonous voice refers to a lack of social skills whereas a voice judged warm and enthusiastic refers to a high level of skills.

A third indicator is named “sentence length” and includes speech rate/pressure, speaking time, and pauses. Dow (1985) observed that “speaking time” and “pauses” were consistent problems for socially anxious individuals in many social interactions. Indeed, monosyllabic speaking (“hmmm,” “yes,” “OK”) will be associated with poor social skills.

Gaze or eye contact is the 4th item on the SPRS. Associated with body orientation, gaze was related to global ratings of anxiety and skills in psychiatric patients (Monti, 1984; Fydrich and Chambless, 1998). A participant who completely avoids or continuously stares at his or her partner will be rated as less socially successful.

Finally, discomfort (5th item) expressed during the interaction through rigid body movements or facial tics, frequent throat clearing, inappropriate laughter, or sarcasm may also indicate low skills. In contrast, relaxed posture and natural body language characterize high social skills.

The SPRS has been validated in a population with anxiety disorders and therefore prone to difficulties in social interaction (Fydrich and Chambless, 1998). It has the advantage of being easily adaptable to all ages and to a wide range of situations (Harb et al., 2003; Lau et al., 2022). The SPRS is short and easy to use, unlike other scales such as the Social Behavior Scale (Trower, 1978). The SPRS is applicable to the observation of videotaped or live conversations between two people. Observers are asked to rate behaviors on a 5-point scale.

The SPRS and the CPS scales are complementary from the point of view of assessing social skills in the sense that the CPS scale focuses on skills that are involved in collaborative problem solving, whereas SPRS assesses more general social skills that are not specific to CPS but that may facilitate it. Thus, they provide both general information about social interactions and specific information about CPS.

#### Translation and cross-cultural validation

As suggested by Wild et al. (2005) in their guide to good practice in translation and cultural adaptation, a first translation should be done from English to French, and then a second from French to English. This method of forward/backward translation allows for quality control to verify consistency between the translation and the original version.

Both scales were translated at our request by Lionbridge (Dublin, Ireland), a company specializing in language and cross-cultural adaptation.

#### Adaptation of scales

In order to verify the comprehensibility and to validate the content of the two annotation scales, we pre-tested them *via* the annotation of six participants (a total of 18 videos, one per collaborative game). The annotations were performed by four annotators, all experts in human behavior (2 psychologists and 2 researchers in Psychology and Human-Computer Interaction).

We measured inter-rater agreement using the intraclass correlation coefficient (ICC, 2 k). This coefficient defines the reliability of ratings by comparing the variability of different ratings of the same individual to the total variation of all ratings and all individuals (Shrout and Fleiss, 1979). In our case, a sample of four raters is selected and they rated all participants. We therefore opted for the ICC two-way random with the mean as the unit of assessment. Meetings between the raters allowed us to perform a qualitative validation of the content (comprehensibility, acceptability, and response modality).

Regarding the CPS scale, the results indicated that the items of the “problem solving” dimension as well as the “Self-evaluation” and “Transactive memory” items of the “collaborative” dimension gave rise to very low inter-rater agreement as well as to responses of the “not applicable” type (Table 2).

**Table 2:** Response not applicable by CPS scale item Shaded items exceeding 50% of data not applicable.

Item	Total of response	Total of no applicable response	Percentage (in %)
“Collaborative” dimension			
Action	72	1	1.39
Interaction	72	2	2.78
Task completion/perseverance	72	2	2.78
Adaptive responsiveness	72	0	0
Audience awareness	72	1	1.39
Negotiation	72	10	13.89
Self-evaluation	72	48	66.67
Transactive memory	72	54	75
Responsibility initiative	72	27	37.5
“Problem solving” dimension			
Organizes	72	36	50
Goal sets	72	36	50
Resource management	72	40	55.56
Flexibility and ambiguity	72	37	51.39
Collects elements of information	72	44	61.11
Systematicity	72	36	50
Relationship	72	38	52.78
Rules “if… then”	72	37	51.39
Hypothesis “what if…”	72	40	55.39

These items were also considered by the annotators to be the most difficult to understand and are specific to one type of CPS (e.g., tasks focused on problem solving and not collaboration). We therefore removed these items from the CPS scale.

Thus, we move from an 18-item scale to a 7-item scale: action, interaction, and perseverance for the participation sub-dimension; adaptive responsiveness and audience awareness for the Perspective taking sub-dimension; and negotiation and responsibility initiative for the Social regulation sub-dimension (Table 3).

**Table 3:** Adaptation of Social skills in collaborative problem solving.

Item	Description
“Collaborative” dimension	
Action	Refers to the skill of participating within the group, whether or not this action is coordinated with other group members The highest score will be given to participants who need very little guidance in the instructions to act in the problem solving. They are comfortable in both familiar and unfamiliar situations
Interaction	Refers to the skill of engaging in verbal and non-verbal behaviors that demonstrate interaction with others The highest level of interaction skills is demonstrated if participants actively initiate coordination efforts, or prompt their collaborators to respond
Task completion/perseverance	Refers to the skill of engaging in the task. The highest score will be given to participants who persist in engagement as indicated by multiple attempts at the problem or by trying different strategies.
Adaptive responsiveness	Refers to the skill of integrating the contributions of collaborators into one’s own thoughts and actions when solving a problem. Participants are able to integrate the contributions of collaborators into their own thoughts and actions. Individuals who rethink the representation of a problem based on evidence reported by a partner demonstrate a high degree of responsiveness
Audience awareness	Refers to the skill of adapting one’s contributions to others by adapting one’s words to the views of others or by making one’s actions visible and understandable to others
Negotiation	Refers to the skill of reaching a resolution or compromise. The participant comments on the ideas of others, offers reasons to support or refute certain statements, negotiates in case of disagreement, and implements consensual solutions after discussion
Responsibility initiative	Refers to the skill of taking responsibility for working on a common representation of the problem, developing a strategic plan toward a solution, and monitoring the group’s progress. This skill also includes asking questions, asking if the other has suggestions, acknowledging the contributions of others and helping to keep the team organized

We also reworked and expanded the wording of the remaining items to fit our study setting and our three collaborative games (see description column in Table 3). For example, we define the “responsibility initiative” item as “the ability to take responsibility for working on a shared representation of the problem, developing a strategic plan toward a solution, and monitoring the group’s progress. This skill also includes asking questions, asking if the other has suggestions, acknowledging the contributions of others, and helping to keep the team organized.” While initially this item was defined as: “Assuming responsibility for ensuring part of tasks are completed by the group.” Since only the “collaborative” dimension remains from the original scale, we will now call the scale the “Social Skills of Collaboration scale” (SSC).

Finally, the annotators reported difficulties in rating the 0–2 scale, which did not allow for precision and discrimination in the evaluation of skills. We therefore made the rating system more quantitative and more homogeneous with the SPRS scale, by changing the rating from 0–2 to a rating of 1–5.

Following preliminary results of scale adaptation indicating good inter-judge agreement and consistency between measurement items and tasks for the SPRS, no structural changes were undertaken (Table 4).

**Table 4:** Social Performance Rating Scale, (Fydrich and Chambless, 1998).

Item	Description
Gaze	(1) Very Poor: Participant completely avoids looking at the partner or stares continually (5) Very Good: Participant keeps eye contact during the conversation, does not stare; shifts focus during pauses and conversation.
Vocal quality	(1) Very Poor: (a) Participant speaks in a flat, monotonous voice; or (b) speaks at a low volume or mumbles; or (c) speaks overly loudly; or has intrusive tone (harsh or unpleasant voice quality) (5) Very Good: Participant is warm and enthusiastic in verbal expression without sounding condescending or gushy.
Conversation flow	(1) Very Poor: Participant makes few attempts to initiate the conversation. Even when prompted by the partner, participant cannot maintain the conversation. Participant uses almost no open-ended questions, or is intrusive in questions and shows no empathy. Participant does not attend to information provided by partner (5) Very Good: Participant easily maintains the conversation and responds smoothly to pauses in the conversation, often by following up on previous information provided by the partner or providing free information about the self on a related topic. Participant introduces new topics fluidly and frequently uses open-ended questions. Participant shows genuine interest in the partner and follows up on the partner’s remarks with warmth or enthusiasm.
Discomfort	(1) Very High: Complete rigidity of arms, legs, or whole body. Constant leg movements or fidgeting with hands, hair, or clothing. Extremely stiff face or constant facial tics. Frequent nervous throat clearing, swallowing, or stuttering. Frequent inappropriate giggling or laughing. Look of extreme discomfort and desire to flee situation shown by 2 or more breaks in role. Participant does not pay attention to the role-play tasks most of the time (5) Very Low: Relaxed body posture and natural body movement. Participant laughs and smiles at appropriate times. S/he shows effective gesturing (to be distinguished from fidgeting). Participant focuses on the task all the time, does not appear at all uncomfortable, but at ease in situation.
Length	(1) Very Poor: Monosyllabic (`hmmm’, ‘yeah’, ‘OK’) speech turns; or responses so long that partner must interrupt or cannot utter reply (5) Very Good: At most times, participant’s utterances are two or more sentences long. Participant acknowledges partner’s remarks without taking over and monopolizing the conversation.

## Validation study

We performed a psychometric evaluation of the newly adapted scales. For this, we assessed each scale’s internal consistency, inter-rater reliability, and structural validity with exploratory factor analysis. We measured convergent and divergent validity using correlations with measures of social anxiety and alexithymia that have been found to impact performance in social interactions (Fydrich and Chambless, 1998; Abe et al., 2020). Finally, we assessed the discriminant validity of our scales by comparing the scores obtained by people with social anxiety and a healthy control group.

### Participants

In this validation study, we recruited 76 participants randomly assigned to 38 dyads (Table 5). Recruitment was done from the general population *via* advertisements on websites and within the university. Eligible individuals were at least 18 years old, without language impairment, and fluent in French.

**Table 5:** Sample demographics.

	Female	Male
N	35	41
Age (in years)	23.46 ± 7.7511 (18, 52)	23.78 ± 7.857 (18, 56)
Education (in years)	14.77 ± 3.273 (12, 24)	14.44 ± 3.091 (9, 20)

### Protocol

Following recruitment, participants completed questionnaires *via* the online platform “Limesurvey”.^1^ These questionnaires allowed us to collect information about the individual characteristics of the participants, e.g., the type of difficulties the participants experience or their emotional states. We will discuss these questionnaires in more detail in the section “measurements.”

Once the questionnaires were completed, we randomly assigned the participants into pairs and invited them to come to the lab to perform the collaborative games. These were the three collaborative games presented in the “Corpus” section: “prisoner’s dilemma,” “survival task” and “invest for college.” The interaction also took place *via* a videoconference platform without any other support than video, thus allowing to collect auditory and visual information in direct interaction conditions.

The participants were given the following general instruction: “During each game, you will have to discuss and collaborate with each other in order to find a common solution to each of the problems you will be presented with.” The experimenter told the participants that their conversation would not be listened to and that they could exchange freely.

#### Measures

The SSC and SPRS annotation scales are available in the Appendices Section. To investigate the impact of interindividual differences on social skills during CPS games, prior to performing the three collaborative tasks, participants completed self-report questionnaires.

The Toronto Alexithymia Scale (TAS-20; Bagby et al., 1994) is the most commonly used scale for assessing alexithymia. This scale is composed of 20 items divided into three dimensions: difficulty identifying emotions, difficulty describing emotions, and outwardly oriented thoughts. The rating is from 1 “complete disagreement” to 5 “complete agreement.”

The Social Interaction Anxiety Scale (SIAS; Mattick and Clarke, 1998) assesses social interaction anxiety, defined as extreme distress in initiating and maintaining conversations with friends, strangers, or potential partners. It is composed of 20 items rated from 0 “not at all representative of me” to 4 “very representative of me.”

The Beck Depression Inventory (BDI; Delay et al., 1963; Beck and Beamesderfer, 1974) assesses the presence of depressive symptoms in participants. This scale consists of 13 items rated from 0 to 3. The higher the score, the greater the depressive symptoms.

#### Annotators and annotation procedure

The annotators were previously introduced to the protocol and the two scales. Among them, two are trained in psychology, one is a clinical psychologist and the last one is an expert in Human-Machine interaction. They received training in annotation and participated in the modifications made to the scales. During this training phase, the ratings of each collaborative game were discussed and discrepancies were resolved. To ensure cohesion and common understanding of the items, the raters met after each annotation to review the interaction videos together and discuss discrepancies between the original ratings and justify their annotation.

To perform their annotations, annotators were given the scales in the form of an Excel spreadsheet with the following instructions, “You will watch videos featuring interactions between two people (one video for each game). After each video, you are to rate the social performance of each participant. Please note that you must watch the video at least twice before you begin your assessment. Once the assessment is complete, you must watch the video again at least once before you can finally validate your answers.”

#### Ethical considerations

This study was approved by the university ethics committee (approval number: CER-Paris-Saclay-2021-060). A written and informed consent and an image right form were signed by all study participants.

Participants could stop their participation in the research at any time and for any reason. In accordance with the provisions of the French Data Protection Act, participants may exercise their rights of access, rectification, or deletion by contacting the project’s scientific director.

Participants completed questionnaires about their mood and personality. They were under no obligation to answer questions that made them feel uncomfortable. Thus, if the participant showed significant emotional changes, the study would be stopped and a professional psychologist would be invited to intervene. In addition, a debriefing with the experimenter (psychologist) was systematically proposed to them at the end of the experiment as well as a relaxation exercise.

### Statistical analysis

Analyses were performed on the mean scores of the three games. A repeated-measures ANOVA showed no significant effect of game on the scores of each item (all *p* > 0.05). Therefore, annotation scores were averaged across the three role-playing games performed by each subject to provide a more reliable assessment of social performance.

Psychometric analysis included assessment of the SSC and SPRS scales’ item characteristics, construct validity, internal consistency, and convergent and divergent validity.

**Figure 2:**
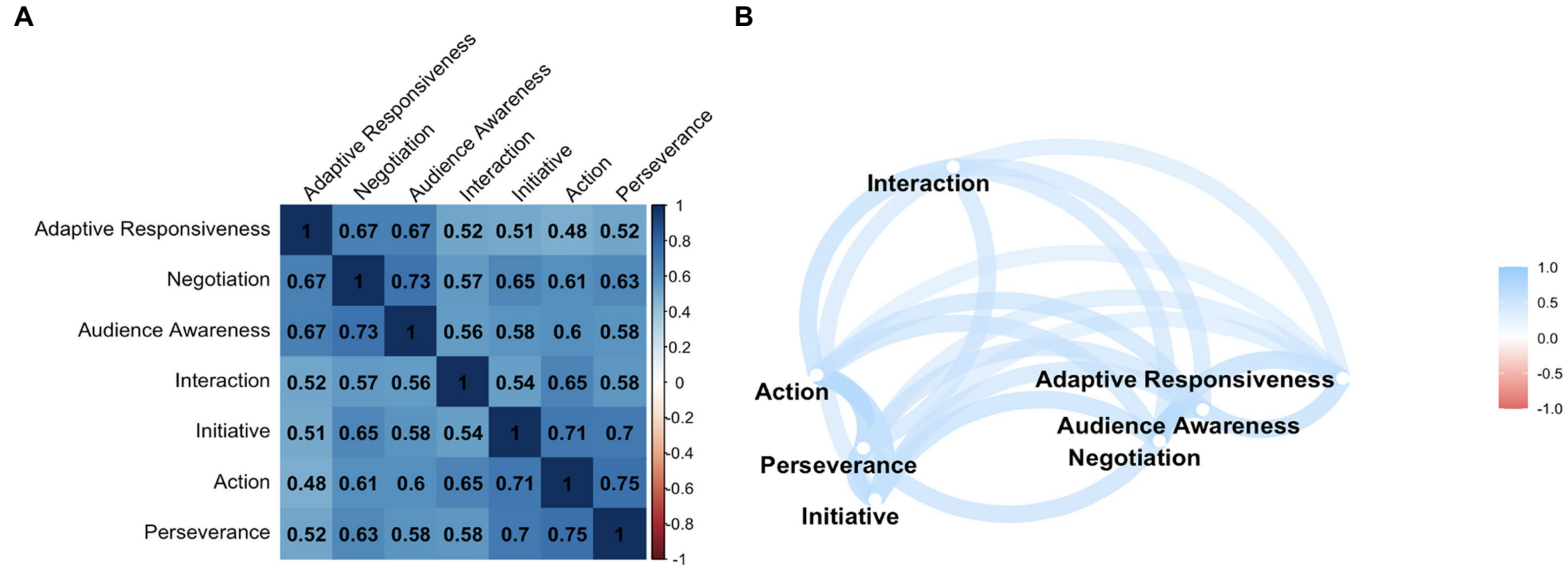
Correlation between items: **(A)** Spearman’s rank correlation coefficients matrix and **(B)** correlation network. Correlation between items: **(A)** matrix of Spearman’s rank correlation coefficients and **(B)** correlation network. The matrix contains the Spearman’s rank correlation coefficients between the 7 items of the SSC questionnaire. Colors indicate the direction and strength of the correlation, with positive correlations displayed in blue and negative correlations in red. Results in bold indicate statistical significance at the *p* < 0.05 level. All results are statistically significant. The correlation network is constructed from all pairwise correlations between items in **(A)**. Items are represented by nodes and are connected by edges. The red and blue lines represent negative and positive correlations, respectively. The color saturation of the line widths is proportional to the strength of the correlation.

Spearman’s correlation coefficients (*rs*) were calculated to examine the homogeneity of the scale (item-total correlation, with a minimum acceptable level of *rs* > 0.3) and to identify whether highly correlated items should be omitted because of redundancy (inter-item correlations, *rs* > 0.9). A correlation network graph was constructed from these results to graphically illustrate the relationships between items (Figure 2B for the SSC scale and 4B for SPRS).

The construct validity (factor structure) of the scales was assessed by exploratory factor analysis (principal factor method with non-orthogonal oblique rotation, also known as oblimin rotation; Costello and Osborne, 2015) to examine underlying concepts and characterize dimensionality. Data were first examined using Bartlett’s test of sphericity (*p* < 0.001 for both scales) and the Kaiser–Meyer–Olkin measure of sampling adequacy (for SSC = 0.90; for SPRS = 0.86), indicating that our sample was appropriate for conducting Exploratory Factorial Analyses (EFA; Beavers et al., 2013). We used a variety of strategies to determine the optimal number of factors to retain, including consideration of the proportion of variance explained for the selected factorial solution, use of Horn’s parallel analysis (Horn, 1965) based on 95th percentile estimation, and calculation of Velicer’s minimum mean partial criterion (MAP) (Velicer, 1976). Finally, items were considered for deletion if their factor loadings were <0.4, or/and if their communalities were <0.3 (uniqueness >0.7).

The reliability of internal consistency (item homogeneity) was assessed by calculating Cronbach’s alpha coefficient (Cronbach, 1951). A coefficient score >0.8 indicates good internal consistency and >0.9 is considered excellent.

Convergent validity was studied using Spearman’s correlation between the total sum of items in each scale and the alexithymia scale. Divergent validity was measured using a correlation with a state anxiety scale and then with a social anxiety scale specific to social interaction.

Finally, discriminant validity was investigated by examining whether the total score of each scale could differentiate a group of interacting anxious participants from a control group. Scores were compared between groups using the nonparametric Kruskal–Wallis test.

Statistical analyses were performed using JASP Version 0.16.1 software (JASP Team, 2022, University of Amsterdam) for descriptive and factor analyses and R 4.1.3 GUI 1.77 High Sierra build (R Foundation for Statistical Computing, Vienna, Austria) using the paran and psych packages for Horn parallel analysis and Velicer’s minimum mean partial correlation for the number of principal components, respectively.

## Results

### Factor structure

#### SSC

We performed a Spearman’s inter-item correlation. The results indicate that all inter-item correlations are positive and significant (Figure 2). The item with the lowest correlations is the “action” item.

Exploratory factor analysis was performed on the 7 items of the SSC scale with an oblique rotation (oblimin).

The one-factor solution had eigenvalues above the Kaiser criterion of 1 and explained 65.4% of the variance. The scree plot was slightly ambiguous and showed inflections that would justify retaining both 1 and 2 factors. Given the sample size, the convergence of the scree plot, and the Kaiser criterion on 1 factor, the one-factor solution was therefore retained in the final analysis. Table 6 presents the factor loadings after rotation. That is, how the 7 items apply to the selected factor.

**Table 6:** SSC’s factor loading.

Item	Factor 1	Uniqueness	Mean	Std.	*S*	*K*
Adaptive Responsiveness	0.738	0.456	4.303	0.587	−0.893	0.878
Negotiation	0.834	0.305	4.216	0.634	−0.756	0.038
Audience Awareness	0.796	0.367	4.194	0.645	−0.714	0.129
Interaction	0.761	0.421	4.351	0.720	−1.303	1.540
Initiative	0.837	0.299	4.376	0.665	−1.129	0.857
Action	0.854	0.270	4.592	0.579	−1.684	2.922
Perseverance	0.836	0.300	4.375	0.683	−1.007	0.442

Std, Standard deviation; *S*, Skewness; *K*, Kurtosis

We observe that all items have a factor load between 0.738 and 0.854. We can also see in Table 6 the “uniqueness” of each variable. Uniqueness is the proportion of variance that is “specific” to the variable and not explained by the factor. The higher the uniqueness, the lower the relevance or saturation of the variable in the factorial model. For example, 45.6% of the variance in the item “Adaptive Responsiveness” is not explained by the factor in the one-factor solution. In contrast, the variance of the “Action” item is relatively small and is not accounted for by the factorial solution (27%).

Thus, the factor analysis of the SSC scale identifies a factor (or domain) composed of 7 items. This factor is related to collaboration and describes the set of social skills necessary for it to take place.

#### SPRS

**Figure 3:**
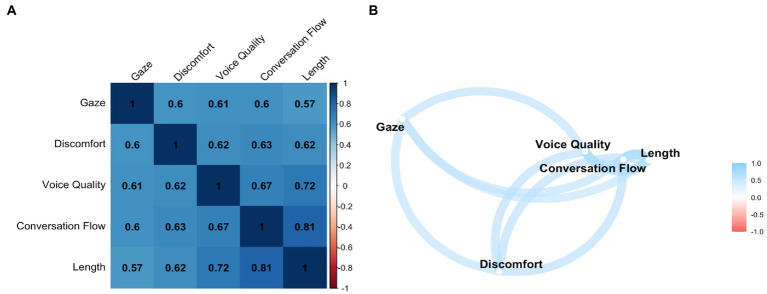
Correlation between items: **(A)** Spearman’s rank correlation coefficients matrix and **(B)** correlation network. Correlation between items: **(A)** matrix of Spearman’s rank correlation coefficients and **(B)** correlation network. The matrix contains the Spearman’s rank correlation coefficients between the 5 SPRS questionnaire items. Colors indicate the direction and strength of the correlation, with positive correlations displayed in blue and negative correlations in red. Results in bold indicate statistical significance at the *p* < 0.05 level. All results are statistically significant. The correlation network is constructed from all pairwise correlations between items in **(A)**. Items are represented by nodes and are connected by edges. The red and blue lines represent negative and positive correlations, respectively. The color saturation of the line widths is proportional to the strength of the correlation.

The Spearman’s correlation performed between the 5 SPRS items also shows significant results and positive relationships (Figures 3A,B).

The five SPRS items also had an EFA with an oblique rotation (oblimin). For the same reasons as the SSC scale, the one-factor solution was preferred to the two-factor solution. This solution explains 67.1% of the variance. The factor loadings are between 0.72 for the “gaze” item and 0.88 for the “length” item (Table 7).

**Table 7:** Factor loading.

Item	Factor 1	Uniqueness	Mean	Std.	*S*	*K*
Gauze	0.720	0.481	4.128	0.908	−1.302	1.312
Vocal quality	0.845	0.286	4.359	0.699	−1.123	0.874
Conversation flow	0.865	0.251	4.260	0.678	−1.114	1.536
Discomfort	0.778	0.395	4.182	0.659	−0.754	0.409
Length	0.876	0.233	4.205	0.701	−0.870	0.813

Std, Standard deviation; *S*, Skewness; *K*, Kurtosis

The factor analysis identified a factor composed of 5 items. This factor corresponds to verbal and non-verbal social skills necessary for communication with others.

### Reliability

For scale validation, inter-rater agreement was measured by calculating the intraclass correlation coefficient (ICC, 2 k). This coefficient defines the reliability of ratings by comparing the variability of different ratings of the same individual to the total variation of all ratings and individuals (Shrout and Fleiss, 1979). In our case, the videos were annotated by at least two raters. Therefore, we opted for the random two-way ICC method with the mean as the unit of evaluation.

The intraclass correlation coefficient ranged from 0 to 1. The results indicate moderate to excellent reliability for the items of both scales. Values below 0.5 indicate poor reliability, values between 0.5 and 0.75 indicate moderate reliability, values between 0.75 and 0.9 indicate good reliability, and values above 0.9 indicate excellent reliability (Koo and Li, 2016).

#### SSC

For the SSC scale, all items obtained moderate to good reliability (0.54 for the “Adaptive Responsiveness” item to 0.80 for the “interaction” item; Table 8).

**Table 8:** Inter-rater reliability for the SSC scale.

Item	ICC 2 K
Adaptive responsiveness	0.54
Negotiation	0.62
Audience awareness	0.66
Interaction	0.80
Initiative	0.70
Action	0.69
Perseverance	0.65

#### SPRS

For the SPRS, all items are between moderate and good reliability (0.50 for the “Discomfort” item to 0.81 for “Gaze” item).

### Internal consistency, convergent, and divergent validity

#### SSC

An overall performance measure was created by summing the raw scores of the seven items. This measure demonstrates excellent internal consistency (Cronbach alpha = 0.928; McDonald omega = 0.929).

**Figure 4:**
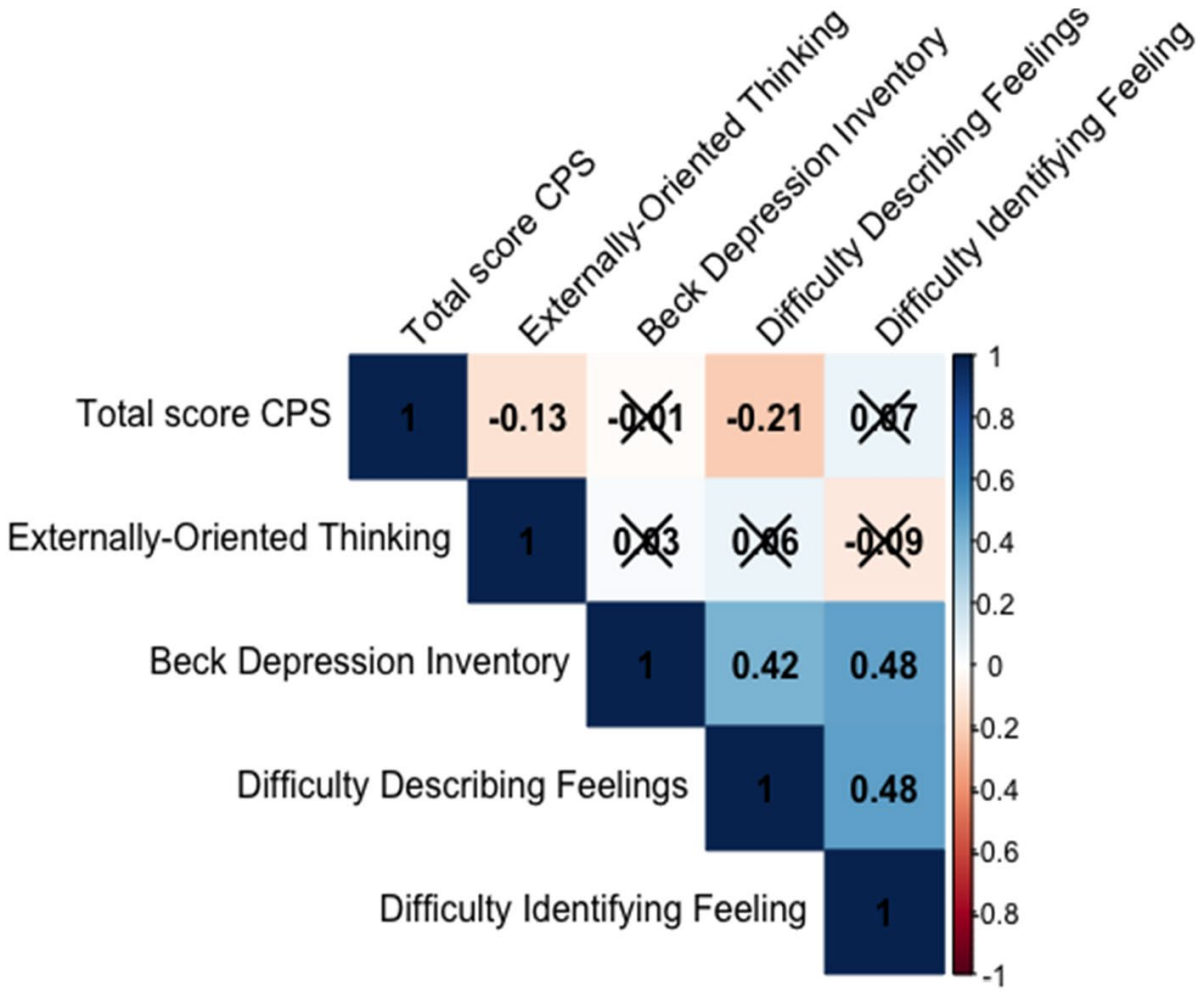
Correlation indicating SSC convergent and divergent validity. Non-significant correlations are marked with an X.

Spearman’s correlations were calculated between the total score and the questionnaire measures. A Bonferroni correction was applied (Figure 4). Good convergent validity was demonstrated by the moderate to large correlations between the total score on the SSC scale and the alexithymia dimensions Difficulty Describing Feelings (TAS DDF *r* = −0.21, *p* = 0.001) and Externally-Oriented Thinking (EOT *r* = −0.13, *p* = 0.05). This indicates that the higher the scores on these dimensions, the less successful the participants are in collaboration.

For divergent validity, a weak correlation between the SSC scale and the BDI depression scale was found (*r* = −0.01, *p* = 0.83; ns).

#### SPRS

An identical approach was taken for the SPRS scale. The scale shows excellent internal consistency (Cronbach alpha = 0.902; McDonald omega = 0.901), higher than that found by Fydrich and Chambless (1998).

**Figure 5:**
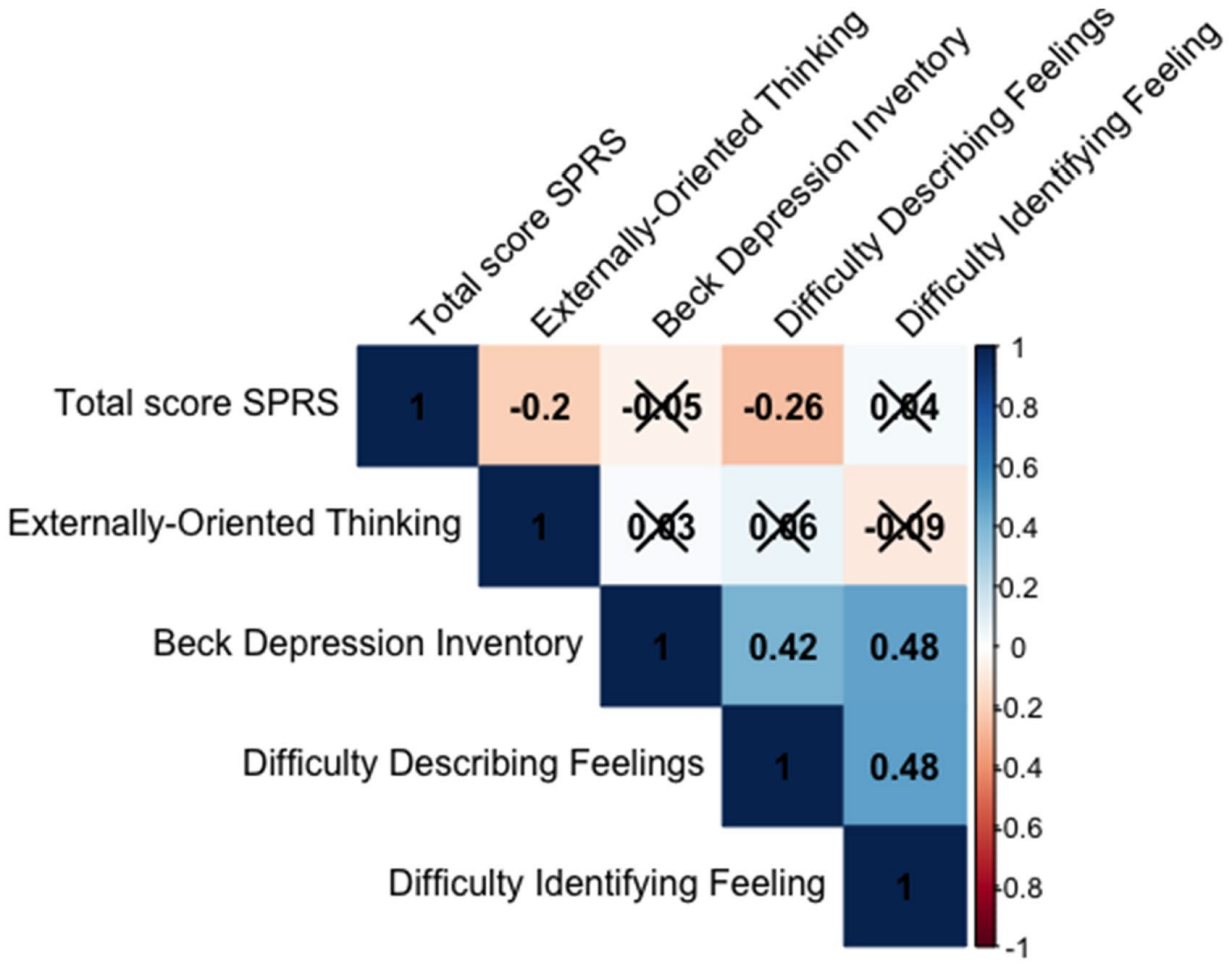
Correlation indicating SSC convergent and divergent validity. Non-significant correlations are marked with an X.

Spearman’s correlations were calculated between the SPRS total score and the alexithymia scale scores (Figure 5). Good convergent validity was also demonstrated by moderate to large correlations between SPRS and alexithymia measures (EOT = −0.20, *p* < 0.001; DDF *r* = −0.26, *p* < 0.001, Bonferroni correction). Participants with higher alexithymia scores had worse performance scores.

Regarding divergent validity, we again found a weak correlation between the SPRS and the BDI depression scale (*r* = 0.05, *p* = 0.15; ns).

### Discriminant validity

Participants completed a questionnaire assessing their level of anxiety in a social interaction context: the Social Interaction Anxiety Scale (SIAS; Mattick and Clarke, 1998). The higher the score, the more anxious symptoms the individuals display. We used the score of 30 as a cutoff value. Thus, participants were divided into two groups based on their SIAS score: control group SIAS <30 and anxiety group SIAS ≥30 (Table 9).

**Table 9:** Demographic description of the sample used for discriminant validity.

Scale	Control	Anxious
N	49	19
Age	23.86 ± 8.49	24.42 ± 6.76
Sex	26 Males	12 Males
Mean SSC	4.51 ± 0.33	4.11 ± 0.44
Mean SPRS	4.35 ± 0.44	4.09 ± 0.46
Mean SIAS	21.47 ± 5.41	38.95 ± 5.43

To assess the discriminant validity of the SSC and SPRS, the total scores of each scale were compared to the SIAS score using a nonparametric Kruskal–Wallis test. It was hypothesized that (a) the SSC and SPRS scores could differentiate anxious from non-anxious individuals and (b) anxious individuals would perform worse than non-anxious individuals.

A review of the SIAS questionnaire responses identified 6 participants with outlier scores that were more than 3 standard deviations from the mean of the distribution. These 6 participants were excluded from the discriminant validity analyses.

#### SSC

Discriminant validity results indicated a tendency for SSC scores to distinguish the anxious group from the non-anxious control group (*p* = 0.06, Bonferroni correction). Anxious individuals performed worse than non-anxious individuals.

#### SPRS

**Figure 6:**
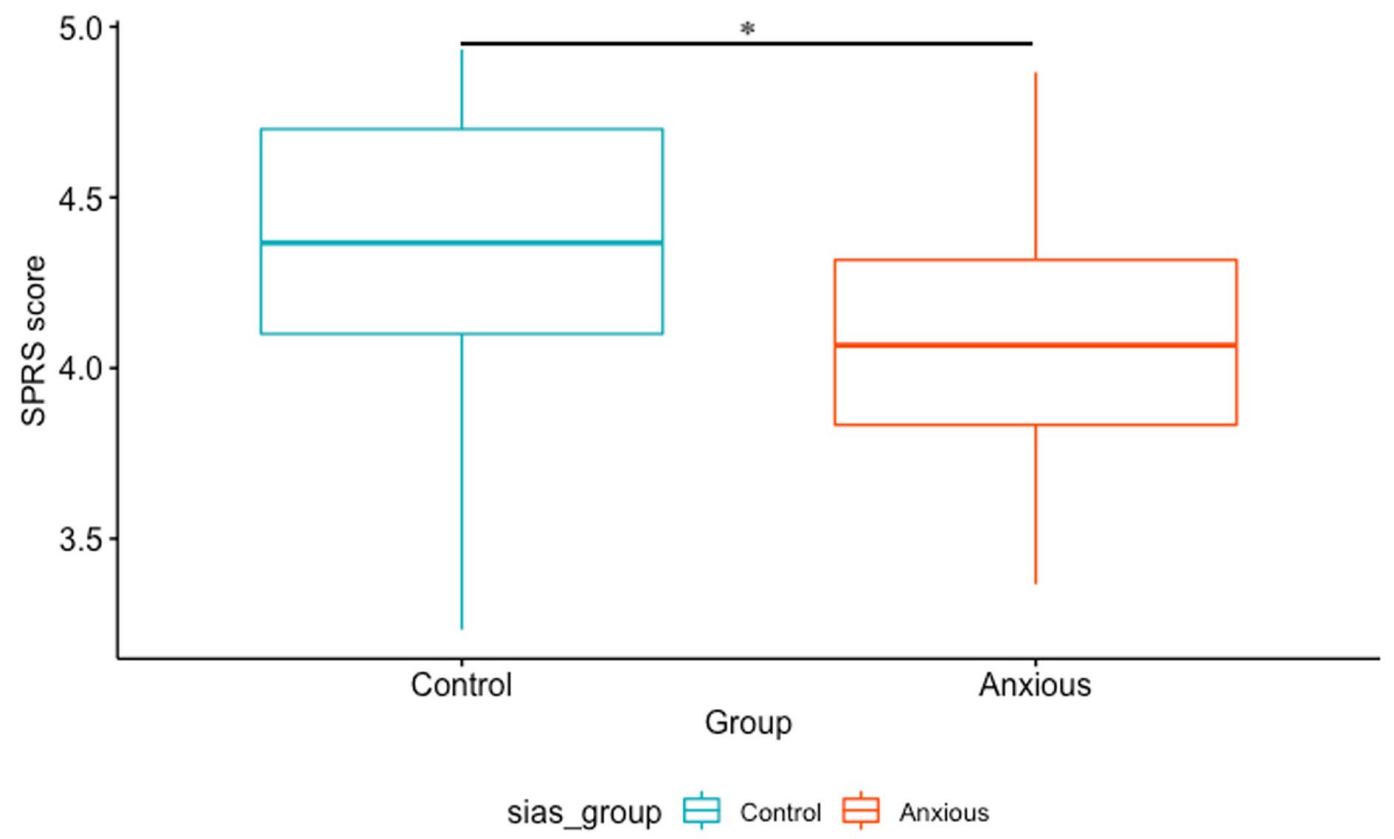
SPRS score according to group (anxious vs. control) ^*^p < 0.05.

The SPRS scores significantly distinguished the anxious group from the non-anxious control group (*p* = 0.03, Bonferroni correction). Anxious individuals were found to perform significantly worse than controls (Figure 6).

## Discussion and conclusion

With its wide applicability to real-life situations, collaborative problem solving can be considered one of the key skills of the 21st century. In this paper, we present the development of a new video corpus of dyadic collaborative interactions and the validation of two scales that allowed the annotation of the social skills involved in these collaborations.

In this new corpus, participants, divided into pairs, were asked to perform three collaborative games in a video conference and without any other support. These dyadic interactions were designed to be as natural as possible and with few environmental and logistical constraints. Thus, we have 228 visual and auditory recordings.

In order to report the level of social skills of the participants, we were confronted with the lack of validated tools to qualitatively assess social skills in a context of unmediated collaboration with others. The Social Skill of Collaboration (SSC) and SPRS were developed to address this lack of tools.

Thus, we constituted the SSC scale on the basis of items derived from existing inventories of CPS skills (Hesse et al., 2015). These inventories were worked on by human behavior experts and seven items were thus retained and reformulated because of their relevance in a collaborative interaction context. The SPRS is a scale for assessing social skills related to communication in social interactions. The English version was translated and adapted to the French population. The original version, judged to be adapted to collaborative interaction contexts, has not been modified except for the translation.

These two scales measure distinct but complementary constructs. Indeed, while the SSC scale assesses social skills specific to the context of collaboration, the SPRS assesses communication skills, useful in all social interaction contexts, that are prerequisites for collaborative skills.

Psychometric analyses confirmed that the SSC and SPRS scales are valid and reliable instruments for measuring social skills. Factor analysis showed a one-factor solution for each of the scales, explaining over 65% of the variance for the SSC scale and 67% for the SPRS. The reliability coefficients of the psychometric analysis showed values that indicated excellent internal consistency (0.93 for the SSC and 0.90 for the SPRS). In contrast to existing tools (O'Neil et al., 2010; Oliveri et al., 2017), both of our scales showed moderate to good inter-rater reliabilities.

The convergent and divergent validity of the SPRS and SSC was confirmed by the pattern of correlations with other reliable and valid measures: correlations with measures of alexithymia (difficulty in identifying emotions and external orientation of thoughts) were significant, whereas those with measures of depression were not. These results are in agreement with those highlighted by Fydrich and Chambless (1998) during the development of the SPRS.

Finally, the two annotation scales allowed us to distinguish between anxious and non-anxious participants. Anxious participants were judged to be less socially successful than non-anxious participants. This result is consistent with difficulties during social interaction put forward in different studies (Monti, 1984; Harb et al., 2003; Stevens et al., 2010).

According to the literature on social skills training (Bellack, 1983; Oliveri et al., 2017), these results seem of particular interest since the development of CPS skills has been defined as essential to professional, academic, and private success. Assessing them with reliable and valid instruments would allow to target training sessions on people’s difficulties but also according to their profile (e.g., alexithymia or anxious people). It would also be possible to follow the progress of the participants throughout the skills training and thus verify its effectiveness.

## Limits and future directions

Although this study established good psychometric properties, several limitations should be noted. One of these includes the relatively small sample size, which limits the generalizability of the results. In addition, when recruiting the sample, we did not target individuals with specific difficulties in social interaction, which may explain the mixed results of the discriminant validation of the SSC scale. A study targeting people with difficulties in social interaction (e.g., anxious or alexithymic people) would allow for further investigation of the discriminative properties of the scale. In addition, psychometric properties such as the ceiling and floor effects should also be investigated in a future study.

Social performance is determined by the evaluation of others (Rose-Krasnor, 1997; Yeates et al., 2007), for this reason, it could be interesting in a principal component analysis to use a larger panel of non-expert annotators. This step will allow us to make our scales more accessible and with generalized psychometric qualities.

The generalizability of these measures is also limited by the strong link between social skills assessment and cultural context (Cavell, 1990; Chapdelaine and Alexitch, 2004; Lane et al., 2013; Grover et al., 2020). Future research is needed to test the measure in different cultural contexts.

While the SSC scale and SPRS were developed in a CPS context, they could be adapted and applied to all types of dyadic social interactions. Further research is needed to extend the current study to other interaction contexts. Nevertheless, the SSC scale and SPRS may be useful to a wide range of researchers interested in assessing social skills.

## Data availability statement

The raw data supporting the conclusions of this article will be made available by the authors, without undue reservation.

## Ethics statement

The studies involving human participants were reviewed and approved by CER-Paris-Saclay-2021-060. The patients/participants provided their written informed consent to participate in this study.

## Author contributions

JHB conducted the study and analyses and wrote the manuscript. J-CM, EP, and CC contributed extensively to the data collection and revision of the manuscript. All authors contributed to the article and approved the submitted version.

## Funding

This research was funded by the French National Research Agency (ANR) within the framework of a TAPAS project (ANR-19-JSTS-0001) https://anr.fr/Projet-ANR-19-JSTS-0001.

## Conflict of interest

The authors declare that the research was conducted in the absence of any commercial or financial relationships that could be construed as a potential conflict of interest.

## Publisher’s note

All claims expressed in this article are solely those of the authors and do not necessarily represent those of their affiliated organizations, or those of the publisher, the editors and the reviewers. Any product that may be evaluated in this article, or claim that may be made by its manufacturer, is not guaranteed or endorsed by the publisher.

## Footnotes

^1^ www.limesurvey.org

## References

[ref1] Abe N. Abe K. Nakashima K. (2020). The role of perceived stress and fear of negative evaluation in the process from alexithymia to over-adaptation. Psychologia 62, 217–232. doi: 10.2117/psysoc.2020-A001

[ref2] Andrews-Todd J. Forsyth C. (2020). Exploring social and cognitive dimensions of collaborative problem solving in an open online simulation-based task. Comput. Hum. Behav. 104:105759. doi: 10.1016/j.chb.2018.10.025

[ref3] Andrews-Todd J. Forsyth C. Steinberg J. Rupp A. (2018). Identifying Profiles of Collaborative Problem Solvers in an Online Electronics Environment. International Educational Data Mining Society, Paper Presented at the International Conference on Educational Data Mining (EDM) (11th, Raleigh, NC, Jul 16-20, 2018).

[ref4] Aram J. D. Morgan C. P. Esbeck E. S. (1971). Relation of collaborative interpersonal relationships to individual satisfaction and organizational performance. Adm. Sci. Q. 16, 289–297. doi: 10.2307/2391901

[ref5] Bagby R. M. Taylor G. J. Parker J. D. A. (1994). The twenty-item Toronto alexithymia scale-II. Convergent, discriminant, and concurrent validity. J. Psychosom. Res. 38, 33–40. doi: 10.1016/0022-3999(94)90006-X, PMID: 8126688

[ref6] Barron B. (2003). When smart groups fail. J. Learn. Sci. 12, 307–359. doi: 10.1207/S15327809JLS1203_1

[ref7] Bause I. M. Brich I. R. (2018). Using technological functions on a multi-touch table and their affordances to counteract biases and foster collaborative problem solving. Int. J. Comput. Support. Collab. Learn. 13, 7–33. doi: 10.1007/s11412-018-9271-4

[ref8] Beavers A. S. Lounsbury J. W. Richards J. K. Huck S. W. Skolits G. J. Esquivel S. L. (2013). Practical considerations for using exploratory factor analysis in educational research. Pract. Assess. Res. Eval. 18, 6

[ref9] Beck A. T. Beamesderfer A. (1974). Assessment of depression: the depression inventory. Mod. Probl. Pharmacopsychiatry 7, 151–169. doi: 10.1159/000395074 4412100

[ref10] Bellack A. S. (1983). Recurrent problems in the behavioral assessment of social skill. Behav. Res. Ther. 21, 29–41. doi: 10.1016/0005-7967(83)90123-7

[ref11] Bellack A. S. (2004). Skills training for people with severe mental illness. Psychiatr. Rehabil. J. 27, 375–391. doi: 10.2975/27.2004.375.391 15222149

[ref12] Bland A. R. Roiser J. P. (2017). Cooperative behavior in the ultimatum game and prisoner's dilemma depends on players' contributions. Front. Psychol. 8:1017. doi: 10.3389/fpsyg.2017.01017, PMID: 28670295 PMC5472703

[ref13] Cavell T. (1990). Social adjustment, social performance, and social skills: a tri-component model of social competence. J. Clin. Child Adolesc. Psychol. 19, 111–122. doi: 10.1207/s15374424jccp1902_2

[ref14] Chapdelaine R. F. Alexitch L. R. (2004). Social skills difficulty: model of culture shock for international graduate students. J. Coll. Stud. Dev. 45, 167–184. doi: 10.1353/csd.2004.0021

[ref15] Cheung M. W.-L. Chan W. (2002). Reducing uniform response bias with Ipsative measurement in multiple-group confirmatory factor analysis. Struct. Equ. Model. Multidiscip. J. 9, 55–77. doi: 10.1207/S15328007SEM0901_4

[ref16] Chezan L. C. Drasgow E. Grybos E. M. (2020). Conversation skills and self-initiated interactions in young adults with autism and intellectual disability. Res. Autism Spectr. Disord. 75:101554. doi: 10.1016/j.rasd.2020.101554

[ref17] Costello A. Osborne J. (2015). Best practices in exploratory factor analysis: four recommendations for getting the most from your analysis. Pract. Assess. Res. Eval., 10, 7. doi: 10.7275/jyj1-4868

[ref64] Creative Problem Solving (2013). Developing Skills for Decision Making and Innovation. London, Routledge.

[ref18] Cronbach L. J. (1951). Coefficient alpha and the internal structure of tests. Psychometrika 16, 297–334. doi: 10.1007/BF02310555

[ref19] Delay J. Pichot P. Lemperiere T. Mirouzee R. (1963). The nosology of depressive states. Results of Beck's questionnaire. The nosology of the depressive states: relations between etiology and semiology: II. Results of Beck's questionnaire. Encéphale. Rev. Psychiatr. Clin. Biol. Thér. 52, 497–504. 14094014

[ref20] Dindar M. Järvelä S. Järvenoja H. (2020). Interplay of metacognitive experiences and performance in collaborative problem solving. Comput. Educ. 154:103922. doi: 10.1016/j.compedu.2020.103922

[ref21] Doggett R. A. Krasno A. M. Koegel L. K. Koegel R. L. (2013). Acquisition of multiple questions in the context of social conversation in children with autism. J. Autism Dev. Disord. 43, 2015–2025. doi: 10.1007/s10803-012-1749-8, PMID: 23292139 PMC3631576

[ref22] Dotson W. H. Leaf J. B. Sheldon J. B. Sherman J. A. (2010). Group teaching of conversational skills to adolescents on the autism spectrum. Res. Autism Spectr. Disord. 4, 199–209. doi: 10.1016/j.rasd.2009.09.005

[ref23] Dow M. G. (1985). Peer validation and idiographic analysis of social skill deficits. Behav. Ther. 16, 76–86. doi: 10.1016/S0005-7894(85)80057-5

[ref24] Frankel A. Gardner R. (2007). Using the communication and teamwork skills (CATS) assessment to measure health care team performance. Jt. Comm. J. Qual. Patient Saf. 33, 549–558. doi: 10.1016/S1553-7250(07)33059-6, PMID: 17915529

[ref25] Fydrich T. Chambless D. L. (1998). Behavioral assessment of social performance: a rating system for social phobia. Behav. Res. Ther. 36, 995–1010. doi: 10.1016/S0005-7967(98)00069-2, PMID: 9714949

[ref26] Graesser A. C. Fiore S. M. Greiff S. Andrews-Todd J. Foltz P. W. Hesse F. W. (2018). Advancing the science of collaborative problem solving. Psychol. Sci. Public Interest 19, 59–92. doi: 10.1177/1529100618808244, PMID: 30497346

[ref27] Greene J. C. (2003). Handbook of Communication and Social Interaction Skills. Psychology Press.

[ref28] Grover R. L. Nangle D. W. Buffie M. Andrews L. A. (2020). “Chapter 1- Defining social skills” in Social Skills Across the Life Span. eds. Nangle D. W. Erdley C. A. Schwartz-Mette R. A. (Elsevier, Inc. Academic Press), 3–24.

[ref29] Hall J. Watson W. H. (1970). The effects of a normative intervention on group decision-making performance. Hum. Relat. 23, 299–317. doi: 10.1177/001872677002300404

[ref30] Harb G. C. Eng W. Zaider T. Heimberg R. G. (2003). Behavioral assessment of public-speaking anxiety using a modified version of the social performance rating scale. Behav. Res. Ther. 41, 1373–1380. doi: 10.1016/S0005-7967(03)00158-X, PMID: 14628786

[ref31] Hesse F. Care E. Buder J. Sassenberg K. Griffin P. (2015). “A framework for teachable collaborative problem solving skills,” in Assessment and Teaching of 21st Century Skills Educational Assessment in an Information Age, 37–56.

[ref32] Horn J. L. (1965). A rationale and test for the number of factors in factor analysis. Psychometrika 30, 179–185. doi: 10.1007/BF02289447 14306381

[ref33] Hutchins E. (1995). Cognition in the Wild. Cambridge, MA, USA: Bradford Books

[ref65] Janssen J. Erkens G. Kirschner P. A. (2012). Task-related and social regulation during online collaborative learning. Metacognition Learning 7, 25–43. doi: 10.1007/s11409-010-9061-5, PMID: 27330520

[ref34] Johnson E. (1994). Auditor memory for audit evidence: effects of group assistance, time delay, and memory task. Audit. J. Pract. Theory 13, 36

[ref35] Kingery J. N. Erdley C. A. Scarpulla E. (2020). “Developing social skills,” in Social Skills Across the Life Span. (London Academic Press: Elsevier), 25–45.

[ref36] Koo T. K. Li M. Y. (2016). A guideline of selecting and reporting intraclass correlation coefficients for reliability research. J. Chiropr. Med. 15, 155–163. doi: 10.1016/j.jcm.2016.02.012, PMID: 27330520 PMC4913118

[ref37] Lane H. C. Hays M. J. Core M. G. Auerbach D. (2013). Learning intercultural communication skills with virtual humans: feedback and fidelity. J. Educ. Psychol. 105, 1026–1035. doi: 10.1037/a0031506

[ref38] Lau N. Zhou A. M. Zhou A. M. Yuan A. Parigoris R. Rosenberg A. R. Weisz J. R. (2022). Social skills deficits and self-appraisal biases in children with social anxiety disorder. J. Child Fam. Stud., 1–12. doi: 10.1007/s10826-021-02194-w PMC10538948 37772042

[ref39] Mattick R. P. Clarke J. C. (1998). Development and validation of measures of social phobia scrutiny fear and social interaction anxiety. Behav. Res. Ther. 36, 455–470. doi: 10.1016/s0005-7967(97)10031-6, PMID: 9670605

[ref40] Monti P. M.. (1984). Multi-modal measurement of anxiety and social skills in a behavioral role-play test: generalizability and discriminant validity. Behav. Assess. 6, 15–25.

[ref41] Oliveri M. E. Lawless R. Molloy H. (2017). A literature review on collaborative problem solving for college and workforce readiness. ETS Res. Rep. Ser. 2017, 1–27. doi: 10.1002/ets2.12133

[ref42] O'Neil H. Chuang S. Baker E. (2010). “Computer-based feedback for computer-based collaborative problem solving” in Computer-Based Diagnostics and Systematic Analysis of Knowledge. eds. Ifenthaler D. Pirnay-Dummer P. Seel N. (Boston, MA: Springer)

[ref43] Ramdhonee-Dowlot K. Balloo K. Essau C. A. (2021). Effectiveness of the super skills for life programme in enhancing the emotional wellbeing of children and adolescents in residential care institutions in a low-and middle-income country: a randomised waitlist-controlled trial. J. Affect. Disord. 278, 327–338. doi: 10.1016/j.jad.2020.09.053, PMID: 32980656

[ref44] Rose-Krasnor L. (1997). The nature of social competence: a theoretical review. Soc. Dev. 6, 111–135. doi: 10.1111/j.1467-9507.1997.tb00097.x

[ref45] Selby E. C. Treffinger D. J. Isaksenr S. G. Lauer K. J. (2004). Defining and assessing problem-solving style: design and development of a new tool. J. Creat. Behav. 38, 221–243. doi: 10.1002/j.2162-6057.2004.tb01242.x

[ref46] Shrout P. E. Fleiss J. L. (1979). Intraclass correlations: uses in assessing rater reliability. Psychol. Bull. 86, 420–428. doi: 10.1037//0033-2909.86.2.420, PMID: 18839484

[ref47] Slof B. Nijdam D. Janssen J. (2016). Do interpersonal skills and interpersonal perceptions predict student learning in CSCL-environments? Comput. Educ. 97, 49–60. doi: 10.1016/j.compedu.2016.02.012

[ref48] Smith-Jentsch K. A. Johnston J. H. Payne S. C. (1998). “Measuring team-related expertise in complex environments,” in Making Decisions under Stress: Implications for Individual and Team Training (Washington, DC, USA: American Psychological Association), 61–87.

[ref49] Spitzer C. Siebel-Jürges U. (2005). Alexithymia and interpersonal problems. Psychother. Psychosom. 74, 240–246. doi: 10.1159/000085148 15947514

[ref50] Stadler M. Herborn K. Mustafić M. Greiff S. (2020). The assessment of collaborative problem solving in PISA 2015: an investigation of the validity of the PISA 2015 CPS tasks. Comput. Educ. 157:103964. doi: 10.1016/j.compedu.2020.103964

[ref51] Stevens M. J. Campion M. A. (1999). Staffing work teams: development and validation of a selection test for teamwork settings. J. Manag. 25, 207–228. doi: 10.1016/S0149-2063(99)80010-5

[ref52] Stevens S. Hofmann M. Kiko S. Mall A. K. Steil R. Bohus M.. (2010). What determines observer-rated social performance in individuals with social anxiety disorder? J. Anxiety Disord. 24, 830–836. doi: 10.1016/j.janxdis.2010.06.005, PMID: 20637563

[ref53] Sun C. Shute V. J. Stewart A. Yonehiro J. Duran N. D'Mello S. (2020). Towards a generalized competency model of collaborative problem solving. Comput. Educ. 143:103672. doi: 10.1016/j.compedu.2019.103672

[ref54] Trower P. (1978). Skills training for adolescent social problems: a viable treatment alternative? J. Adolesc. 1, 319–329. doi: 10.1016/S0140-1971(78)80035-9, PMID: 756425

[ref55] Trower P. (1980). Situational analysis of the components and processes of behavior of socially skilled and unskilled patients. J. Consult. Clin. Psychol. 48, 327–339. doi: 10.1037/0022-006X.48.3.327, PMID: 7381092

[ref56] Velicer W. F. (1976). Determining the number of components from the matrix of partial correlations. Psychometrika 41, 321–327. doi: 10.1007/BF02293557

[ref57] Wild D. Grove A. Martin M. Eremenco S. McElroy S. Verjee-Lorenz A.. (2005). Principles of good practice for the translation and cultural adaptation process for patient-reported outcomes (PRO) measures: report of the ISPOR task force for translation and cultural adaptation. Value Health 8, 94–104. doi: 10.1111/j.1524-4733.2005.04054.x, PMID: 15804318

[ref58] Yeates K. O. Bigler E. D. Dennis M. Gerhardt C. A. Rubin K. H. Stancin T.. (2007). Social outcomes in childhood brain disorder: a heuristic integration of social neuroscience and developmental psychology. Psychol. Bull. 133, 535–556. doi: 10.1037/0033-2909.133.3.535, PMID: 17469991 PMC2841002

[ref59] Zhuang X. MacCann C. (2008). Development and Validity evidence supporting a teamwork and collaboration assessment for high school students. ETS Res. Rep. Ser. 2008, i–51. doi: 10.1002/j.2333-8504.2008.tb02136.x

[ref60] Zimmerman B. Schunk D. (2011). “Self-regulated learning and performance: an introduction and an overview,” in Handbook of Self-Regulation of Learning and Performance. eds. Zimmerman B. J. Schunk D. H. (Routledge/Taylor & Francis Group), 1–15.

