# Supplementary material for: Adaptation and validation of two annotation scales for assessing social skills in a corpus of multimodal collaborative interactions
Source: Front Psychol. 2022 Oct 27;13:1039169. doi: 10.3389/fpsyg.2022.1039169 (PMC9648354; doi:10.3389/fpsyg.2022.1039169)
Supplement: Supplementary file 1 [file Data_Sheet_1.PDF]

SOCIAL SKILLS OF COLLABORATION SCALE IN FRENCH

Item	Item description
**Action**	Aptitude à participer au sein du groupe, que cette action soit ou non coordonnée avec les autres membres du groupe.<br>De (1) aptitude très faible<br>À (5) aptitude très élevée
**Interaction**	Aptitude à avoir des comportements verbaux et non-verbaux qui démontrent une interaction avec les autres.<br>De (1) aptitude très faible<br>À (5) aptitude très bien
**Achévement des tâche / persévérance**	Aptitude à s'engager et à persister dans son engagement comme indiqué par de multiples tentatives dans la résolution du problème ou en essayant différentes stratégies.<br>De (1) aptitude très faible<br>À (5) aptitude très élevée
**Réactivité adaptative**	Aptitude à intégrer les contributions des collaborateurs dans ses propres pensées et actions lors de la résolution de problème.<br>De (1) aptitude très faible<br>À (5) aptitude très élevée
**Sensibilisation du public**	Aptitude à adapter son comportement pour qu'il convienne mieux aux autres :<br>Il s'agit de la capacité à adapter ses contributions aux autres en adaptant ses propos aux points de vue des autres ou en rendant ses actions visibles et compréhensibles pour ses collaborateurs.<br>De (1) aptitude très faible<br>À (5) aptitude très élevée
**Négociation**	Aptitude à parvenir à une résolution ou à un compromis. Le participant commente les idées des autres, offre des raisons pour soutenir ou réfuter certaines affirmations, négocier en cas de désaccord et met en œuvre des solutions consensuelles après discussion.<br>De (1) aptitude très faible<br>À (5) aptitude très élevée
**Initiative de responsabilité**	Aptitude à prendre la responsabilité de travailler sur une représentation commune du problème, de développer un plan stratégique vers une solution et de suivre l'avancement du groupe. Cette aptitude comprend également le fait de poser des questions, de demander si l'autre a des suggestions, de reconnaître les contributions des autres et d'aider à maintenir l'organisation de l'équipe<br>De (1) aptitude très faible<br>À (5) aptitude très élevée

SOCIAL PERFORMANCE RATING SCALE, ADAPTION IN FRENCH

Item	**Item description**
**Regard/ contact visuel**	Inclut le contact visuel ou le regard dans la direction de l'autre sans nécessairement établir un contact visuel<br>De (1) très mauvais : Lorsque le participant évite complètement de regarder son partenaire ou le fixe continuellement<br>À (5) très bien : Lorsque le participant maintient un contact visuel pendant la conversation, ne fixe pas ; déplace l'attention pendant les pauses et la conversation.
**Qualité vocale**	Inclut les catégories de qualité sonore, de hauteur, de clarté et de volume<br>De (1) très faible : Lorsque le participant parle d'une voix plate et monotone ; ou parle à faible volume ou marmonne ; ou parle trop fort, ou a un ton intrusif (qualité de voix dure ou désagréable).<br>À (5) Très bien : Lorsque le participant parle d'une voix chaleureuse et enthousiaste sans paraître condescendant ou exubérant. Il ne parle pas trop fort, ou avec un trop faible volume.
**Longueur**	Recouvre le « rythme/la pression de la parole », le « temps de parole » et les « pauses»<br>De (1) Très faible : Lorsque le prise de parole du participant est monosyllabique (« hmmm », « oui », « OK »); ou des réponses si longues que le partenaire doit les interrompre ou ne peut pas répondre.<br>À (5) Très bien : la plupart du temps, les déclarations du participant se composent de deux phrases ou plus. Le participant prend acte des remarques de son partenaire sans pour autant prendre le relais et monopoliser la conversation.
**Inconfort**	Incluent les « mouvements des extrémités », « les auto-manipulations » (par exemple se toucher le visage ou les mains), « l'expression du visage », la « posture » et les « gestes »<br>De (1) Très élevé : rigidité complète des bras ou de tout le corps. Agitation des mains, des cheveux ou des vêtements. Visage extrêmement raide ou tics faciaux constants. Raclements de gorge nerveux fréquents, déglutition ou bégaiement. Rires ou sarcasmes inappropriés fréquents. Apparence d'inconfort extrême et désir de fuir la situation manifestée par deux interruptions ou plus du rôle. La plupart du temps, le participant ne prête pas attention aux tâches du jeu de rôle.<br>À (5) Très faible : posture du corps détendue et mouvement naturel du corps. Le participant rit et sourit aux moments appropriés. Il ou elle fait des gestes efficaces (à distinguer de l'agitation). Le participant se concentre sur la tâche en permanence, ne semble pas du tout mal à l'aise, mais au contraire à l'aise dans la situation.

**Flux de conversation**	Comprend les éléments d'une déclaration personnelle appropriée ainsi que la prise de parole, l'intérêt pour le partenaire et le suivi de la conversation.<br>De (1) Très faible : Le participant fait peu de tentatives pour initier la conversation. Même lorsqu'il y est invité par le partenaire, le participant ne peut maintenir la conversation. Le participant n'utilise presque pas de questions ouvertes, ou il est intrusif dans les questions et ne montre aucune empathie. Le participant ne tient pas compte des informations fournies par le partenaire.<br>À (5) Très bien : Le participant maintient facilement la conversation et réagit avec souplesse aux pauses de la conversation, souvent en reprenant les informations précédentes fournies par le partenaire ou en fournissant des informations libres sur lui-même sur un sujet connexe. Le participant introduit de nouveaux sujets de manière fluide et utilise fréquemment des questions ouvertes. Le participant montre un véritable intérêt pour le partenaire et donne suite à ses remarques.
